# Gesneriads, a Source of Resurrection and Double-Tolerant Species: Proposal of New Desiccation- and Freezing-Tolerant Plants and Their Physiological Adaptations

**DOI:** 10.3390/biology12010107

**Published:** 2023-01-10

**Authors:** Ane Legardón, José Ignacio García-Plazaola

**Affiliations:** Department of Plant Biology and Ecology, University of the Basque Country (UPV/EHU), Barrio Sarriena s/n, 48940 Leioa, Spain

**Keywords:** resurrection species, desiccation tolerance, freezing stress, Gesneriaceae, oxidative stress, structural damage

## Abstract

**Simple Summary:**

In the current scenario of climate change, plants need to overcome a great amplitude of temperatures and increasingly common droughts in the very same space. Gesneriads are a worldwide family of plants in which many “resurrection” species have arisen: plants with the ability to withstand long periods of time with very little water content and successfully revive upon water availability. Due to their rocky and mountainous habitat, many of them have to face a great temperature variability and freezing temperatures, and indeed, their resurrection ability and freezing tolerance share similar metabolic responses. Tolerance of gesneriads to different environmental stresses is thought to be applicable in crop growth improvement, but their difficult indoor cultivation and outdoor accessibility are major obstacles for their study. Therefore, this review aims to identify common patterns in the already known resurrection species to propose new tentative resurrection gesneriads, as well as gather the metabolic responses to desiccation and freezing stress as a way of making them more reachable to the scientific community.

**Abstract:**

Gesneriaceae is a pantropical family of plants that, thanks to their lithophytic and epiphytic growth forms, have developed different strategies for overcoming water scarcity. Desiccation tolerance or “resurrection” ability is one of them: a rare phenomenon among angiosperms that involves surviving with very little relative water content in their tissues until water is again available. Physiological responses of desiccation tolerance are also activated during freezing temperatures, a stress that many of the resurrection gesneriads suffer due to their mountainous habitat. Therefore, research on desiccation- and freezing-tolerant gesneriads is a great opportunity for crop improvement, and some of them have become reference resurrection angiosperms (*Dorcoceras hygrometrica, Haberlea rhodopensis* and *Ramonda myconi*). However, their difficult indoor cultivation and outdoor accessibility are major obstacles for their study. Therefore, this review aims to identify phylogenetic, geoclimatic, habitat, and morphological features in order to propose new tentative resurrection gesneriads as a way of making them more reachable to the scientific community. Additionally, shared and species-specific physiological responses to desiccation and freezing stress have been gathered as a stress response metabolic basis of the family.

## 1. Introduction

Gesneriads are a family of pantropical middle-sized plants comprising about 150 genera of perennial herbs, shrubs, and small trees. This family has been reported to include over 3400 species, but the number grows everyday thanks to phylogenetic studies [1]. The richness and dimension of the family have enabled “resurrection” or desiccation-tolerant (DT) plants to thrive. This characteristic relies on the plant’s ability to withstand very low water contents in their tissues (~10% relative water content (RWC)) and fully recover upon re-watering, a very rare phenomenon among angiosperms, with less than 0.1% of them being DT [2]. 

Most DT gesneriads are relict species that have been sheltered in mountainous habitats, usually in northern-oriented limestone areas, which means that they have had to overcome drought and cold stress, which forced double adaptation [3]. In fact, tissue desiccation is the consequence of both drought and freezing stresses; therefore, similar physiological mechanisms are triggered in response to both, to the point that desiccation tolerance could entail some kind of cross-tolerance to freezing stress. Moreover, the cross-tolerance that resurrection characteristics confer has been postulated to have biotechnological applications in crop growth [4].

The geographical distribution and diversification of gesneriads are exceptionally advantageous in terms of finding and studying new double-tolerant species, but their scattered and difficult-to-reach habitats present an accessibility obstacle. Additionally, their slow growth and complex environmental conditions are disadvantageous for indoor growth. Consequently, most of the few studies on resurrection gesneriads have been performed within the native area of the species [5]. Identifying common phylogenetic, geoclimatic, habitat, and morphological characteristics of the already known resurrection gesneriads should help in finding new species in more convenient locations and widen the knowledge about their double tolerance.

## 2. Species Identification

### 2.1. Phylogeny

**Figure 1:**
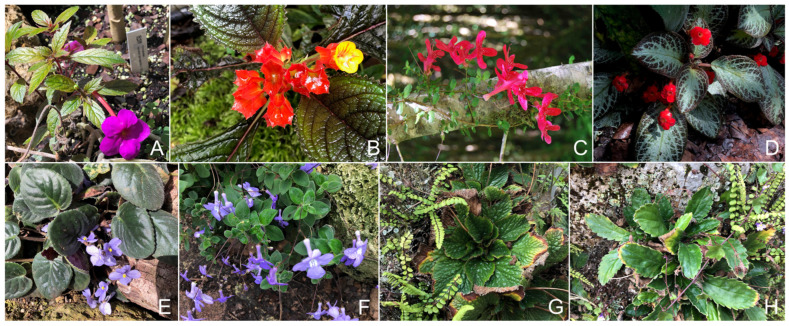
Examples of plants species from Gesnerioideae (**A**–**D**) and Didymocarpoideae (**E**–**H**): *Achimenes* sp. (**A**), *Alloplectus* sp. (**B**), *Asteranthera ovata* (**C**), *Episcia* sp. (**D**), *Streptocarpus* spp. (**E**,**F**), *Ramonda myconi* (**G**), and *Haberlea rhodopensis* (**H**).

The Gesneriaceae family has colonized a great diversity of habitats and developed specialized plant–animal interactions that have led to a great diversity of floral morphologies as well (Figure 1). These traits were first used for phylogenetic species classification, but because they highly converged in different Gesneriaceae lineages, the early taxonomy of the family was complex and contradictory [6,7,8]. 

In recent years, the family has undergone a deep reconstruction due to advancements in molecular-phylogenetic studies, which concluded with a new basis for Gesneriaceae phylogeny in 2013 [1]. Nevertheless, there are limited genetic sites that provide information for phylogenetic characterization of the family; therefore, phylogenetic relationships in some genera remain unclear, or there is no consensus regarding them [9]. 

Nowadays, the Gesneriaceae family contains three subfamilies:

- Sanangoideae, which did not officially become a subfamily until 2013, when it was defined as a monotypic family (*Sanango racemosum*) endemic to South America. It has a distinctive globose and slightly four-partite ovary with a depression in the internal structure and on top, from which the style arises surrounded by a large cupular disc [1].
- Gesnerioideae, which was initially named New World (NW) Gesneriaceae, since it was thought to contain only neotropical species. However, nowadays it also contains gesneriads from Asia and Australia, and it is considered a heterogeneous group with a well-established phylogeny [1,10]. It is generally characterized by the presence of seed endosperm, two equally sized cotyledons with limited growth, a nectary with separated glands, and an inferior ovary [11].
- Didymocarpoideae, which has been typically recognized as Old World (OW) Gesneriaceae; in this case, with the exception of *Rhynchoglossum azureum*, species of this subfamily are indeed found in Asia, Africa, and Europe [12,13]. The intrinsic morphological characteristics of the subfamily include the lack of endosperm, unequal cotyledon growth, ring-shaped nectary, and superior ovary [11].

Even though prior phylogenetic classification established nine different tribes for Gesnerioideae, nowadays it only contains five: Gesnerieae, Titanotricheae, Napeantheae, Beslerieae, and Coronanthereae. Gesnerieae is the “core tribe” that has included the four obsolete tribes and has become the largest one. Current classification is strongly supported by molecular data, and despite the shift in tribe number, Gesnerioideae phylogeny has remained quite stable throughout the years [1].

Didymocarpoideae is divided into two tribes: Epithemateae and Trichosporeae. Species in Epithemateae are thought to be relicts from an enormously diversified group that suffered a number of extinctions, which resulted in a few isolated genera with unusual and marked morphological characteristics. Trichosporeae has typically been the larger and more complex tribe, giving rise to uneven large groups. This has entailed a deep reconstruction of the subfamily that is still in process, allowing more homogeneous species classification [1,13,14,15]. Some genera have been newly established (*Billolivia*, *Michaelmoelleria*, *Chayamaritia*, *Glabrella*, *Microchirita*, *Middletonia*, *Rachunia*, *Somrania*) or recovered (*Dorcoceras*, *Loxocarpus*), while others have gained species from other genera (*Damrongia*, *Oreocharis*, *Loxostigma*, *Deinostigma*, *Paraboea*, *Primulina*, *Streptocarpus*)*,* lost species by relocation to other genera (*Boea*), or lost species by synonymization (*Acanthonema*, *Hovanella*, *Colpogyne*, *Linnaeopsis*, *Nodonema*, *Schizoboea*, *Briggsia*) [13,16,17,18,19,20,21,22,23,24,25,26,27,28]. 

**Figure 2:**
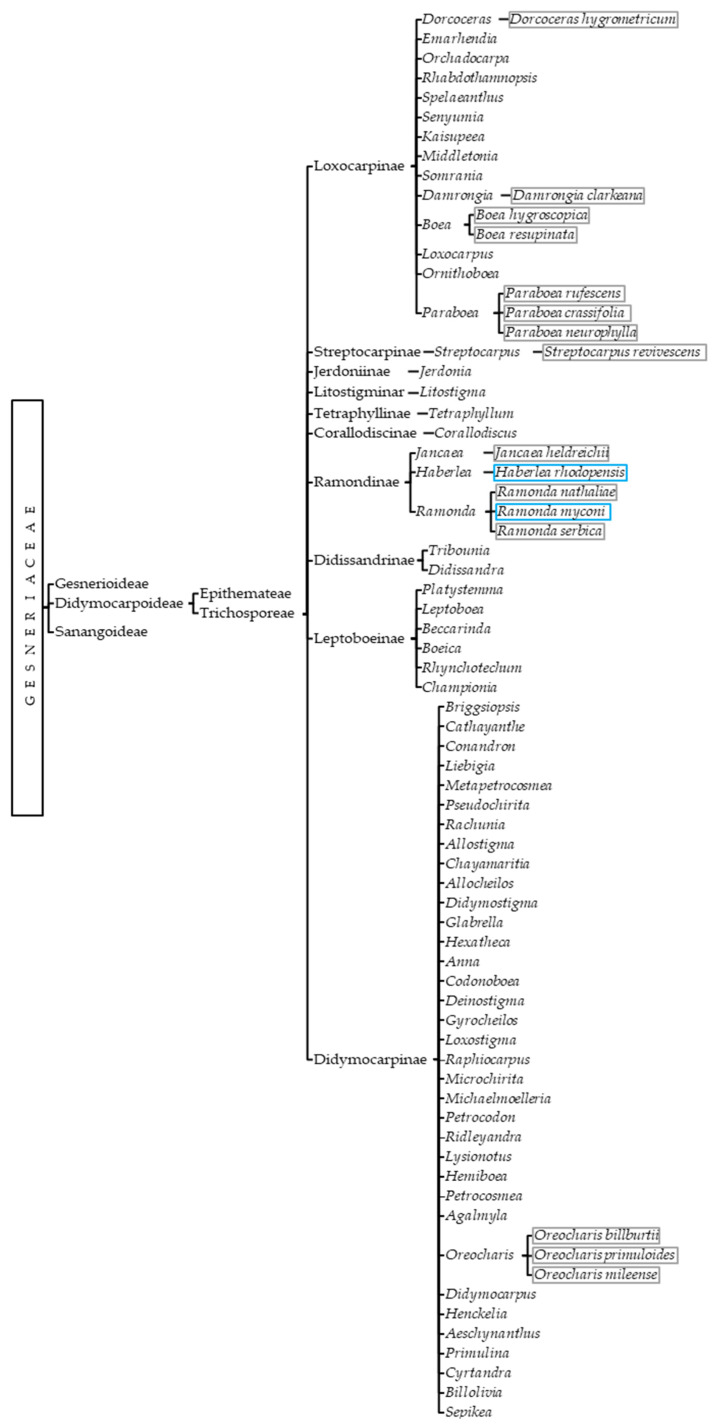
Current phylogenetic classification of Didymocarpoideae with resurrection (grey squares) and double-tolerant (blue squares) species.

All documented DT gesneriads are members of the Trichosporeae tribe; thus, efforts should be focused on untangling its phylogeny. Known resurrection species are *Dorcoceras hygrometricum*, *Damrongia clarkeana*, *Boea hygroscopica, Boea resupinata*, *Paraboea crassifolia*, *Paraboea rufescens, Paraboea neurophylla, Streptocarpus revivescens*, *Jancaea heldreichii*, *Ramonda serbica*, *Ramonda nathaliae*, *Ramonda myconi*, *Haberlea rhodopensis*, *Oreocharis billburttii*, *Oreocharis primuloides,* and *Oreocharis mileensis* [3,29,30,31,32,33,34,35,36,37,38,39]. Even if *Henckelia* and *Corallodiscus* still have no known resurrection species, it has been proposed that they potentially contain them [1]. When it comes to freezing tolerance, *Ramonda myconi* and *Haberlea rhodopensis* are the only resurrection gesneriads that have been tested for freezing stress, but due to geographic, habitat, and morphologic similarities, many of them may indeed be double-tolerant [40,41]. On top of that, the homogeneity of the latest phylogenetic classification implies that Trichosporeae genera already containing DT species could be sources of new resurrection plants (Figure 2).

### 2.2. Geographic Distribution and Habitat

#### 2.2.1. Origin and Geographic Evolution of Gesneriads

The most supported hypothesis regarding the origin and evolution of gesneriads proposes that ancestors of Gesneriaceae probably originated in the neotropics in South America during the Late Cretaceous, with possible points of origin in the temperate Andes and Amazonian rainforest. Indeed, the disjunctive geographic distribution of the Coronanthereae tribe on the Pacific coasts of South America, Australia, New Zealand, and some islands in between and its current placement in the Gesnerioideae subfamily imply a NW origin of Gesneriaceae and posterior dispersal to the OW. The split between Didymocarpoideae and Gesnerioideae dates from 44.7 Mya in the Late Palaeocene/Early Eocene and would have occurred though multiple independent long-distance oceanic dispersals from South America to the Indian Plate via Antarctica until 45 Mya, when it collided with the Eurasian Plate [42,43,44]. The basal taxa of the OW are considered to be Jerdonia (mountains of southwest India), Corallodiscus (Himalayas and China), and Tetraphyllum, Leptoboea, and Boeica (Himalayas and adjacent areas) [8,10]. 

Dispersal to Europe apparently happened quite rapidly after the collision between the two tectonic plates [10]. Quaternary glaciations led to changes in the elevation and latitudinal distribution of species, and three glacial refugia were defined in the Iberian, Italian, and Balkan Peninsulas [45,46]. Other hypotheses suggest that such species already existed in the refugia in the Tertiary, prior to the Quaternary oscillations [47]. However, such hypotheses would need phylogenetic clarification regarding the age estimation of the subtribe [45]. Independent of their origin, just five species (all of them DT) inhabit Europe: four in the Balkan Peninsula (Jancaea heldreichii, Ramonda serbica, Ramonda, nathaliae, and Haberlea rhodopensis) and a single species in the Pyrenees (Ramonda myconi) [48]. Transgression of the species to Africa from Eurasia followed that to Europe, and they could have first colonized Madagascar, from where individuals were dropped to Africa via the land bridge that existed 25–35 Mya [10].

#### 2.2.2. Current Distribution and Habitat

Currently, gesneriads are mainly distributed in tropical and subtropical regions of Asia, Africa, and South and Central America, and they predominantly grow on rocks or trees of mountain forests; meanwhile, they are scarce on lowlands [1,43]. 

In the NW, conformed by Sanangoideae and Gesnerioideae, they primarily became diverse in the northern Andes and Central America and then in the West Indies and the Brazilian Atlantic forest [49,50,51]. Tropical and subtropical Asia are the main distribution areas of Didymocarpoideae, containing 85% of the genera and 90% of the species. 

In addition, the Indo-China Peninsula, Southwest China, and nearby limestone regions are diversity centers of the subfamily [52]. 

Most DT and freezing-tolerant (FT) gesneriads described so far occur in the northern hemisphere [38]. They are found sheltered in the Pyrenees, the Balkan Peninsula, and central and northern China [10,13]. Resurrection gesneriads in temperate areas in the northern hemisphere principally inhabit rocky surfaces with very little soil coverage that have served as ecological shelters for these species [53,54]. They are usually limestone karst with high porosity and salinity in fragmented landscapes [12,55].

Epiphytism and lithophytism have arisen extensively in gesneriads. Epithytic growth is more extensively spread in NW gesneriads, especially in the Columneinae clade, and lithophytism in OW temperate zone gesneriads, although it is prevalent in both areas. Epiphytism and lithophytism do not directly trigger speciation in gesneriads, but they do promote a lower extinction rate and a greater diversification rate [1,8]. These habitats force species to survive with low nutrient and water availability, high irradiance, and wide temperature ranges. The rapid water flow does not allow the plant to absorb water and generates hydric stress, even in wetter climates [56]. The fluctuating environmental conditions of these habitats, such as water availability, have enabled a great diversity of plants to thrive. Furthermore, the adverse environmental conditions of rocky habitats are favorable for vascular resurrection plants, which become the dominant species in this habitat. The scarce water availability coupled with the low temperatures of the mountainous habitat make these suitable places to find double-tolerant species [42,43].

Species that no longer undergo desiccation stress might have lost this characteristic in favor of greater growth rate and plant size. However, in some species such as Boea hygroscopica, which is endemic to wet forest understories and never experiences seasonal drought, this characteristic has been maintained, implying that a greater range of habitats could be considered when looking for resurrection species [2].

**Figure 3:**
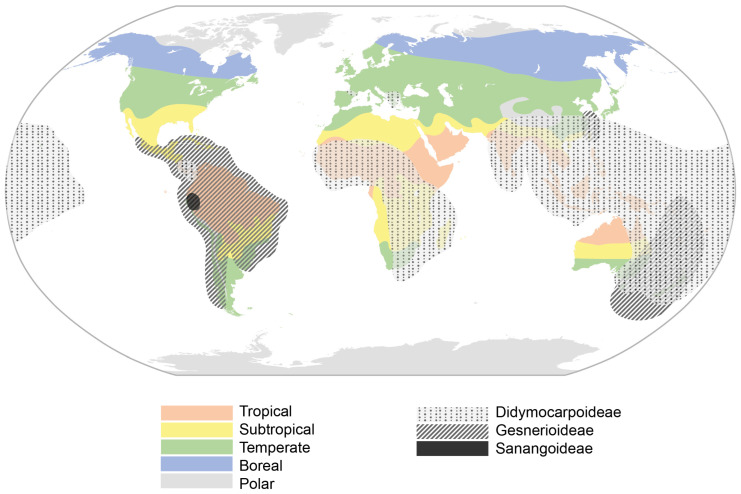
World climatic areas and distribution of the three Gesneriaceae subfamilies.

Nevertheless, DT Didymocarpoideae species have arisen in arid areas that are mostly subjected to freezing temperatures in mountains of the OW. These habitats can also be found in the NW Andes, where hypothetically the Gesnerioideae subfamily arose and is maintained. However, no DT Gesnerioideae has been found so far. There have been some examples of independently developed rhizomatous species in the OW, but they have been mainly developed in NW Gesnerioideae, and scaly rhizomes and tubers can only be found there [3]. General climatic areas seem to have partly modelled the appearance of DT species, since they dominate temperate areas of the northern hemisphere and even some subtropical areas in Africa and China. Therefore, aridity and freezing temperatures themselves do not seem to be enough to trigger them (Figure 3). 

### 2.3. Morphological Characterization

#### 2.3.1. Adaptation Pressures

Different speciation pressures that influence niche occupation have been identified during recent decades. Climatic conditions and oscillations play a major role in this, but morphology and growth forms have been proposed as the main drivers of habitat colonization, with their influence also being notable in gesneriads [57,58]. 

Plant morphology is also shaped by the life habits of the plant. To a large extent, gesneriads are small-sized plants; therefore, they may have needed to develop epiphytic and lithophytic growth forms to occupy sunnier niches. This adaptation would have required drought-resistant leaf morphology, organs, or strategies even in wetter environments. Indeed, most gesneriads (even shrubs) are either epiphytic or lithophytic, and even non-resurrection gesneriads show drought adaptations in their leaf morphology and arrangement. Thus, we hypothesize that morphological plasticity and preadaptation to drought were developed early in the family, making it possible for plants to conquer different habitats and climates worldwide.

The process of leaf curling and folding has obvious ecophysiological advantages for resurrection species, but at the same time it generates enormous tension that has to be efficiently channeled to avoid the generation of mechanical and structural damage. At both the macro and micro scale, leaves have to fold and unfold efficiently following cycles of hydration and dehydration. This mechanism has been studied in detail in the spikemoss *Selaginella lepidophylla* [59]. In the case of angiosperms, several papers individually link specific morphological traits to desiccation and cold tolerance, but an integrated view of the morphological response as a whole is still missing.

The habitats of DT gesneriads in temperate zones tend to be rocky surfaces in cold mountainous areas, where many times they are often exposed to direct sunlight. Therefore, they have to overcome great temperature variation, high irradiance, and drought. Thermal tolerance, both cold and hot, is greatly influenced by the height, leaf morphology, and growth form of the plant [60,61]. 

#### 2.3.2. Morphological Desiccation and Freezing Tolerance Traits

Annual life habit is a drought avoidance strategy, as plants have a high growth intensity that enables them to complete their life cycle during optimal environmental conditions. Therefore, desiccation tolerance is a mechanism that has been developed in perennial plants, as they thoroughly invest multiple metabolic and physiological resources into growing resistant aboveground organs [62,63]. Annual species are rare among gesneriads, with some species scattered in mainly perennial genera in subtropical and tropical areas, while perennial herbs are the predominant growth form [3].

As desiccation tolerance has been mainly found in slow-growing small plants, it has been suggested that it trades off with growth and reproduction [64]. In parallel, these characteristics have been related to the resilience they offer: the slow growth and small size give the plant control over its metabolic reactions with more precision than non-resurrection plants, so they can use resources in a more efficient way according to environmental fluctuations [65]. European gesneriads (*Ramonda* spp., *Haberlea rhodopensis*, and *Jancaea heldreichii*) have been reported to have long-lived leaves and slow growth [36]. These characteristics can surely be extrapolated to other temperate zone DT gesneriads that are potentially FT due to their habitat (*Jancaea heldreichii*, *Ramonda serbica*, *Ramonda nathaliae,* and *Dorcoceras hygrometricum,* among others).

Leaf morphology and disposition are also important. Many species exposed to freezing temperatures are small, acaulescent, and flat and form compact rosettes, while taller plants are usually associated with freeze avoidance mechanisms [66]. Smaller plants use tolerance strategies, which confer resistance to colder temperatures [67]. Tolerance of rosette growth is associated with the insulation that the rosette structure itself provides, as it reduces the loss of heat from the leaves [68]. Furthermore, flat rosettes placed at ground level can take advantage of the ground heat output during cold nights. In addition to height, leaf area also decreases with elevation, which minimizes plant exposure to cold air [67,69]. Meanwhile, leaf dry matter content and thickness increase, which allows better resource conservation in the plant and the creation of densely packed rosettes with coarse leaves. These traits are appropriate for cold acclimation, but also for dry environments and high temperature variation. This seems to be true for temperate and artic alpine zones, but in Afroalpine, Andean, and Hawaiian areas, plants can develop very different heights with freezing tolerance [67,68,70,71]. Nevertheless, gesneriads do seem to follow this trend, and flat and dense rosettes with small leaves are found in all European species, many Chinese and Himalayan genera (e.g., *Corallodiscus*), and African *Streptocarpus*, among others [3]. Furthermore, work on *Streptocarpus* shows that the rosette growth form can increase diversification because of the deep shade adaptation it provides [17,45].

Small leaf area is usually related to denser leaf venation [72]. Leaf veins are responsible for mechanical support, molecule transport, and water flux due to transpiration [73,74,75]. Thus, greater leaf vein density has been postulated to confer drought resistance in leaves and whole plants [72]. Plants with small leaves and considerable vein density would typically inhabit drier and more exposed places, contrary to plants with bigger leaves and lower vein density, which would occupy wet and shady areas [76,77]. Vessel diameter also influences freezing and desiccation tolerance. However, a balance is needed between obtaining vascular efficiency with wide conduits and preventing freezing- and desiccation-induced embolism by using narrow veins [78]. In the case of drought-induced embolism, smaller pore size of the pit membrane is an important trait to consider. In the case of freezing stress, narrow veins are the most essential feature [79]. It is also important to note that although leaves with higher primary vein density are less prone to major vein cavitation, they are more susceptible to minor vein cavitation. Thus, high primary vein density and low minor vein density would be the most suitable combination [80]. High major vein density and small leaves are redundant mechanisms that contribute to more efficient water transport, which would aid in more easily resolving blockages caused by xylem embolisms under drought and freezing stress and help protect the vein system from damage [81]. Venation typology and density in gesneriads have not been researched in terms of function in counteracting environmental stressors, and there seems to be no record of venation patterns of different genera. However, it has been found that temperate zone DT gesneriads exposed to freezing temperatures mostly share a dense reticulate/cross-venulate venation pattern with conspicuous veins. These traits seem to be less pronounced in DT gesneriads in other habitats and tend to show more longitudinal patterns. 

Trichomes, which make the leaves even coarser, also play an important role, as they participate in the protection against biotic and abiotic stresses, such as extreme temperature variations (both low and high), light intensity, drought, and high ultraviolet radiation [82]. At high temperatures, trichomes accelerate heat loss by reflecting sunlight and increasing the convection and thermal conductivity of leaves [83]. Lower leaf temperatures also help minimize transpiration rates for better water use efficiency in dry conditions [84]. Accumulated phenolic compounds in non-glandular trichomes are capable of filtering UV and high visible radiation [85]. They can even store glutathione against oxidative stress [86]. On the other hand, glandular trichomes are capable of synthesizing and excreting secondary metabolites such as phenolics. These trichomes can serve as morphological adaptations under low temperatures, and they play a part in decreasing photoinhibition [87,88]. Some plants, such as *Solanum habrochaites*, increase their trichome density when exposed to high temperatures; *Caragana korshinskii*, when exposed to scarce precipitation; *Arabidopsis thaliana* mutants, when exposed to UV-B; and *Betula pendula*, under freezing stress [88,89,90,91]. This supposes not only a constitutive but also an inducible origin of trichomes. Trichomes are widespread in gesneriads, which gives a hint about the preadaptation of the family to overcoming environmental stressors and the tendency to be denser in DT species from temperate zones. They are commonly simple and uniseriate, and their characterization is often used for species identification [3].

When it comes to leaf margins, dentated and serrated edges have higher photosynthetic activity. It has been hypothesized that greater vein density and free-ending veins that go from the central vein to the margin, which is a characteristic of toothed leaves, increase hydration, transpiration, and gas diffusion and decrease boundary layer resistance [92,93]. Therefore, they allow better photosynthetic performance under dry conditions. Indeed, vein patterns associated with dentated margins seem to be the decisive characteristic that indicates greater performance [93]. Studies with both serrated and non-serrated leaves from *Juniperus* spp. have associated toothed-margin species with drier habitats, narrower veins, and lower risk to the xylem. Moreover, smooth margins are usually farther from secondary veins and thus are more hydraulically vulnerable [94].

Additionally, dentated and serrated edges, widely developed features in gesneriads, have been proposed to facilitate the folding of leaves and the rapid rehydration and shape recovery [95]. In the same way, tissues from the petioles are arranged in semi-circular cross-sections, which, along with the dense leaf venation, are highly suitable for directing internal forces during the critical points of dehydration and rehydration; in some cases, this morphological trait is visible in the external form of the petiole. Indeed, Kampowski et al. describe the contraction of the petiole as one of the first noticeable morphological signs of drought stress in *R. myconi* [96].

**Figure 4:**
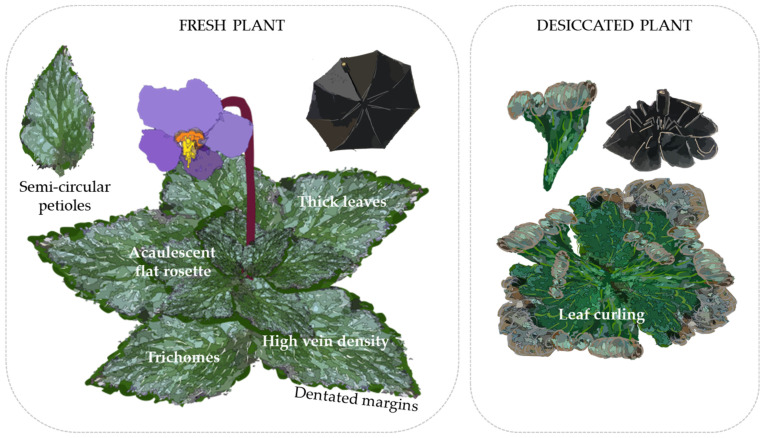
Morphological preadaptations of gesneriads to desiccation. A foldable umbrella is used as an analogy of the structural requirements for a successful process of leaf curling.

As a result, gesneriads growing in mountainous regions need to overcome a great variety of extreme conditions, which leads to a complex relationship among morphological features. Thus, temperate zone DT and FT gesneriads have been found to be perennial, slow-growing, small acaulescent plants with flat compact rosettes. Plant compaction involves a small leaf area with great dry matter content per area and thickness, which, along with trichomes, gives the plant a coarse touch. High vein density and narrow veins are also indispensable for hydraulic and mechanical support, the latter aided by a petiole in semi-circular cross-section and dentated margins (Figure 4). However, this pattern does not entirely adjust for species in areas susceptible to drought that lack freezing stress, as they exhibit more subtle morphological characteristics: they tend to have less compact acaulescent rosettes with longer and less coarse leaves (possibly due to lower dry matter content) and more longitudinal vein patterns. Therefore, differentiation between resurrection and non-resurrection morphology is not always clear, and the morphology described for double tolerance will be mainly considered for species proposal.

When it comes to the Gesnerioideae–Didymocarpoideae dichotomy, morphological differences primarily signify different flower colors and subsequent pollination syndrome. Gesnerioideae are dominated by the transition of any other color to red, and Didymocarpoideae are based on bidirectional transitions between white and purple, which has positively influenced Gesnerioideae diversification, but not extinction rates. This diversification is directly related to the hummingbird diversification that occurred in the NW [8]. Apart from that, no clear morphological difference has been documented between the two major subfamilies. Nevertheless, it seems that intrinsic evolutionary, climatic, or metabolic drivers make Didymocarpoideae more prone to developing desiccation tolerance.

Nevertheless, desiccation tolerance is not the only growth form that gesneriads have evolved to overcome drought stress. Alternative strategies would comprise leaf abscission and geophytism. Leaf abscission, which is confined to Streptocarpus genera, consists of an ever-growing large leaf that, under unfavorable climatic conditions, creates an abscission line from which the dry part of the leaf is detached. Geophytism is present in both Indo-China and America, with a much greater prevalence in Gesnerioideae. In fact, rhizomes can be found in both main subfamilies, but scaly rhizomes and tubers only in Gesnerioideae [3]. Rhizomes and tubers have been developed in tropical and subtropical areas, presumably with a marked seasonality that confers environmental adverse conditions in certain periods of the year. In contrast, resurrection gesneriads are mainly found in temperate or subtropical mountainous areas where adverse conditions frequently occur. Both strategies perfectly fit the environmental conditions they face: it would be biologically inefficient to constantly sprout new plants from rhizomes in temperate mountainous areas, and the resurrection strategy seems to be too expensive for a limited period of the year. However, desiccation tolerance and geophytism are not mutually exclusive strategies and have been found in the same species of pteridophytes and some angiosperms, such as Pitcairnia burchellii, Chamaegigas intrepidus, and Eragrostis invalida [38,97,98,99]. In Pitcairnia burchellii, the resurrection strategy is activated and the rhizomes act as storage organs during water stress. When the plant suffers a very long period in the desiccated state, starch is mobilized from the rhizomes, and the production of new tissue is prioritized instead of the recovery of the desiccated tissue, which indicates that intermediate mechanisms could also exist in gesneriads [97].

### 2.4. Tentative Desiccation/Double-Tolerant Species

The identified phylogenetic, geographic, and morphological patterns can serve as support for identifying tentative resurrection and FT species prior to physiological studies. Here, we propose tentative double-tolerant/DT gesneriads, so that greater availability of these species could help expand our understanding of the DT and FT characteristics in angiosperms (Table 1).

## 3. Physiological Adaptations for Desiccation and Freezing Tolerance 

### 3.1. Desiccation Tolerance Strategies among Gesneriads

Desiccation tolerance has been described in at least nine Gesneriaceae genera, implying that this is the angiosperm family with the largest number of DT genera [38]. So far, mechanisms have been characterized among a significant number of studies, and in fact two gesneriads (*D. hyrgrometricum* and *H. rhodopensis*) are among the top five most studied resurrection angiosperms [5]. Both species, together with *R. serbica* and, to a lesser extent, others such as *P. rufencens, P. crassifolia*, *O. mileensis*, *R. myconi,* and *R. nathaliae*, have allowed researchers to unravel the mechanisms of desiccation tolerance in gesneriads [34,39,100,101,102]. Even though more studies are needed, currently available information suggests that most resurrection gesneriads share similar protective mechanisms, although minor exceptions to this general rule were reported in a comparison between *D. hygrometricum* and *H. rhodopensis* [103].

In response to desiccation, all resurrection gesneriads utilize a strategy known as homoiochlorophylly; that is, they retain chlorophyll (Chl) in the desiccated state. The opposite strategy, shown by some monocots, is poikilochlorophylly, which involves a complete degradation of the photosynthetic apparatus. However, in gesneriads, Chl retention is not always complete; for example, it has been reported to decrease by 20 to 70% in the desiccated state in *Ramonda* species [104]. Parallel to Chl loss, there is a reduction in associated genes, such as those of Chl-a/b-binding antenna proteins [105]. Most other photosynthetic proteins are largely maintained during desiccation, so there is no need to resynthesize them upon rehydration. 

The rate of CO_2_ assimilation is remarkably low in *R. myconi,* and it decreases concomitantly with the loss of water content in *H. rhodopensis*. This process is first due to stomatal closure and afterwards to reduced photochemical activity [101,106]. In parallel, there is a shift from linear electron transport to alternative pathways such as PSI-dependent cyclic electron flux. However, ATP has not been detected in *H. rhodopensis* in the full dry state, which could be due to its massive use for energy-dependent metabolic reactions. This switch between linear electron flow (LEF) and cyclic electron flow (CEF) may contribute to the absence of a drastic decrease in the NADP/NADPH ratio, although a slight increase in NADPH could be observed at 38% water content [107]. Once rehydration starts, the activity of PSI is more rapidly recovered than that of PSII, while the PSI/PSII ratio, D2 protein, and LHCI and LHCII apoproteins (Lhca1 and Lhcb2, respectively) remain stable [106]. These observations suggest that the partial loss of Chl does not indicate the occurrence of damage in the photosynthetic apparatus. Furthermore, given that Chl molecules are a major source of reactive oxygen species (ROS) when photosynthesis is impeded, partial Chl degradation represents the activation of a photoprotective mechanism [108]. As a consequence of the potential ROS generation within the photosynthetic apparatus, oxidative stress is one of the main challenges associated with the process of desiccation and subsequent rehydration [109].

At the same time, the physiological responses conferred by the resurrection strategy have been favorable for colonizing cold mountainous habitats, and what started as a cross-tolerance to freezing stress has evolved into a double tolerance. 

### 3.2. Avoiding Reactive Oxygen Species Formation

To counteract ROS production, resurrection gesneriads constitutively express large pools of antioxidant molecules, including ascorbate and glutathione [102,110]. Other low-molecular-weight antioxidants are phenolic compounds (phenolic acids, polyphenols), whose biosynthetic route (Shikimate pathway) is overexpressed during desiccation in *H. rhodopensis* [105]. A similar polyphenol composition has been described in all gesneriad species studied so far: *H. rhodopensis*, *R. myconi*, *R. serbica*, and *D. hygrometricum* [111,112,113]. Thus, Georgieva et al. proposed that the high polyphenol content is a characteristic feature of gesneriads [114].

The activity of all of these antioxidants is complemented by the action of antioxidant enzymes. For example, Gechev et al. found more genes in *H. rhodopensis* encoding superoxide dismutases, monodehydroascorbate reductases (MDHARs), and glutathione reductases than in most plant species with sequenced genomes, which gives a hint about its constitutive tolerance against desiccation [115]. Mladenov et al. also reported on the accumulation of ascorbate peroxidase and glutathione peroxidase in response to desiccation, as was shown in *R. Nathalie*. The antioxidant machinery that accumulates during desiccation is also maintained in the desiccated state to be used during the first stages of subsequent rehydration. Thus, during the critical first hours of rehydration, superoxide dismutase, catalase, glutathione reductase, and glutathione S-transferase activity remains high to overcome oxidative stress [102,103,104,105,106,107,108,109,110,111,112,113,114,115,116].

One of the main targets of ROS is polyunsaturated fatty acids, whose oxidation gives rise to the formation of lipid peroxides that propagate in membranes through peroxidation chains. Paradoxically, polyunsaturated fatty acids enhance membrane fluidity in desiccated tissues. This is probably why the response of the unsaturation ratio varies so widely among the gesneriads studied: in *H. rhodopensis,* it was not affected by desiccation, while it increased in *B. hygroscopica* and decreased in *R. serbica* [117,118,119]. The main antioxidant involved in avoiding the propagation of such peroxidation chains is tocopherol, and in fact the enhancement of this antioxidant in response to desiccation is a general trait observed in most (if not all) DT plants, including gesneriads such as *D. hygrometricum*, *R. myconi*, and *H. rhodopensis* [101,109,118,120].

The production of ROS within the photosynthetic apparatus can be prevented by simply reducing light absorption within the photosystem antennae. To reduce photon absorption by Chl, *H. rhodopensis* constitutively expresses high levels of red anthocyanins in the abaxial side of the leaves [41]. When the leaves curl, the anthocyanic layer becomes exposed to light, causing a decrease in light reaching the mesophyll. Another strategy to reduce ROS formation is to enhance the dissipation of energy absorbed by Chl as heat, so-called non-photochemical quenching (NPQ). This mechanism is linked to the accumulation of certain proteins, such as PsbS, and the presence of zeaxanthin [121]. PsbS is specifically induced by desiccation in *H. rhodopensis,* while zeaxanthin has been shown to be synthesized in response to desiccation in *R. myconi* [101,122].

### 3.3. Avoiding Structural Damage

A second challenge linked to desiccation is mechanical/structural damage caused by cell shrinkage, which is an obvious consequence of desiccation and leaf curling. First of all, cell walls have to be flexible enough to allow correct folding and to follow such volume alterations [123]. Changes in cell wall permeability and plasticity have been reported in *H. rhodopensis* [124]. Indeed, Mladenov et al. reported downregulated levels of genes involved in lignin and cellulose synthesis and increased levels of enzymes involved in cell wall remodeling. The plasticity of the cell wall mainly depends on the relationship between the cellulose–xyloglucan network and pectin polysaccharides, whereby changes in their composition and connection lead to changes in cell flexibility [116].

In *H. rhodopensis*, the primary central vacuole disappears when the cell desiccates, and smaller secondary vacuoles emerge in the vicinity of the cell wall. At the same time, organelles take the place of the central primary vacuole [110]. The increase in the number of smaller vacuoles, which have a greater area/volume ratio, makes it possible to maintain the membrane surface area during the volume reduction caused by water loss [125]. Desiccated chloroplasts (termed desiccoplasts) adopt a rounded shape, but grana are kept intact during desiccation, although their repeat distance is diminished due to the shrinkage. The most noticeable change in desiccoplasts is the enhancement in the number and size of plastoglobules [110]. 

Maintaining membrane integrity is the main objective of desiccation tolerance strategies. This is accomplished by profound lipid remodeling, and membrane stabilization is further strengthened by the interaction with proteins, sugars, and remaining water molecules [109]. Consequently, the degradation of lipids is a limited phenomenon among resurrection plants, and membranes are highly preserved. In chloroplasts of *R. myconi*, membrane stability is enhanced by a partial conversion of monogalactosyldiacylglycerol (MGDG) to digalactosyldiacylglycerol (DGDG), a bilayer-forming lipid [101]. Stability is reinforced in *H. rhodopensis* by the presence of a dense luminal substance (DLS), most likely a phenolic compound that prevents conformational changes of thylakoids [126]. Additionally, phospholipids, such as phosphatidylethanolamine and phosphatidylcholine, are degraded during desiccation, and there is an increase in phospholipase D [105]. The accumulation of other lipids, such as sitosterol, is also a species-specific response to desiccation in *H. rhodopensis* [118].

The accumulation of compatible solutes, acting as osmoprotectants, is a general response to water loss. In *D. hygrometricum*, *R. myconi*, and *H. rhodopensis*, sucrose is the main compound accumulated in response to desiccation [37]. Sucrose can be produced after starch degradation or can come from gluconeogenesis, as there is consumption of glycolytic intermediates directly related to the accumulation of sucrose [110,118]. In *D. hygrometricum*, raffinose family compounds also increase during desiccation [103]. At the later stages of desiccation, the massive accumulation of sucrose is more directly related to membrane protection by preventing non-bilayer phase separation and membrane fusion [109]. Coincident with their protective functions, both sucrose and raffinose sharply decline after rehydration [110,118].

Once the water potential surpasses a certain threshold (−100 MPa), there is a process of vitrification, and the cytoplasm reaches the so-called glassy state, an amorphous metastable state [109]. In this situation, most metabolic activities cease, and chemical reactivity is inhibited. Transition to the glass state implies positive aspects for DT organisms, as the diffusion of oxygen is greatly reduced, decreasing ROS generation and preventing further water loss. Vitrification was studied in *R. myconi* by Fernández-Marín et al., who showed that this stage can occur in nature, as it can be reached in desiccated leaves at 20 °C [40].

### 3.4. Cellular Protection

Desiccation induces the expression of genes encoding several sets of protective proteins. This is the case of early light-induced proteins (ELIPs) and PsbS, which are involved in the maintenance of chloroplasts and the regulation of photosynthesis. Their expression is maintained during the rehydration process, and some of them are still present 7 days after the onset of rehydration. Desiccation also induces massive expression of late embryogenesis abundant (LEA) proteins in *H. rhodopensis*, *R. serbica,* and *D. hygrometricum* [115,116]. These proteins act as water replacement molecules to maintain the structure of the membranes and organelles, as they have little possibility of interacting with other molecules due to their low capacity for forming hydrogen bonds [127]. 

Damaged DNA is another key aspect of maintaining genome integrity. It can be repaired by several processes, including nucleotide excision repair, in which genes have been reported to be specifically activated during dehydration in *H. rhodopensis*. In case cellular damage occurs, autophagy is also an option [105]. By inducing autophagy, cell death can be inhibited in order to recycle damaged structures to create new structures needed for the protective response of the organism against water deficiency stress, as has been observed in *D. hygrometricum* [120]. The promotion of autophagy is in accordance with the decreasing transcription and protein accumulation of AMC4 in *H. rhodopensis* during desiccation and works as a promoter of programmed cell death under stress [116,128]. This is how DT plants suppress senescence when dehydrated [109]. 

Overall, with the limited information currently available, it is known that most DT mechanisms are common to all resurrection gesneriads. However, there are some others that seem to be species-specific. Thus, specific features such as ascorbic acid and glutaric acid accumulation during desiccation were found only in *D. hygrometricum,* while DNA repair and increased β-aminoisobutyric acid and β-sitosterol were found in *H. rhodopensis*. When DT mechanisms in gesneriads are compared with those in other homoiochlorophyllous angiosperms, such as *Craterostigma*, the same conclusion is reached: in essence, the tolerance strategies are similar, with only minor species-specific differences [103]. 

### 3.5. Freezing-Induced Desiccation

More than half a century ago, Kappen demonstrated that desiccated leaves of *R. myconi* were able to successfully recover after immersion in liquid nitrogen [129]. Recently, the same phenomenon of recovery from freezing conditions was re-examined in resurrection gesneriads in both field and laboratory studies [40,101,106,130]. Cross-tolerance to freezing and desiccation is somewhat logical, since dehydration prevents the formation of ice crystals inside cells. In fact, it has been documented that *H. rhodopensis* has the ability to dehydrate rapidly under freezing temperatures, and this could be interpreted as a freezing tolerance mechanism [130]. Such rapid desiccation might be mediated by the presence of narrow epidermal channels of the leaves [131]. Consequently, most responses are shared between drought-induced desiccation (DID) and freezing-induced desiccation (FID) [132]. However, there are slight differences between these two processes; for example, FID is characterized by faster recovery of PSII compared to DID [133]. In addition, during FID, secondary vacuoles are reported to appear at 60% RWC. This process is similar to the one that happens after DID, but it occurs more rapidly after FID, most likely because of the RWC recovery rate enabled by the environment and the influence of freezing [132].

In addition to FID, it has been documented in manipulative experiments that hydrated leaves of *R. myconi* freeze at a relatively high temperature (−2 °C) [40]. Tissue freezing involves an abrupt reduction in photochemical efficiency (Fv/Fm), which correlates with the enzymatic formation of zeaxanthin. Interestingly, the glass transition in hydrated leaves occurs at −15 °C, meaning that enzyme activity is possible in frozen leaves of *R. myconi* [40]. This justifies the induction of antioxidant enzymes in frozen leaves of *H. rhodopensis* [130]. Recovery of Fv/Fm is initially fast upon transfer to warm conditions and is completely restored in 6 h. This means that even in winter, *R. myconi* is photosynthetically active whenever the temperature is above the freezing point [101].

Apart from the obvious fact that desiccation protects the plant from ice formation, in order to fully withstand winter stress, resurrection gesneriads have to develop a profound metabolic reconfiguration through a process of seasonal acclimation. Only a few studies have addressed the specific mechanisms that *H. rhodopensis* and *R. myconi* employ to cope with low-temperature stress. These include substantial induction of thermal energy dissipation, which is in agreement with a high accumulation of zeaxanthin and the PsbS protein [101,106]. Other stress proteins that accumulate in *H. rhodopensis* in response to low temperature are Lhcb5, Lhcb6, dehydrins, and ELIPs [132]. Genes encoding lipocalins are also upregulated [41]. Lipocalins are small ligand-binding proteins that can be found in both the cell and the chloroplast membrane. In winter, there is also a substantial accumulation of low-molecular-weight metabolites, such as polyphenols [130]; sugars, such as trehalose, maltose, raffinose, sucrose, and glucose; amino acids, such as proline, glycine, serine, alanine, asparagine, and aspartate; polyamines, such as putrescine and ornithine; and antioxidants of the glutathione–ascorbate system [41,101,122,130]. The joint action of all of these mechanisms decreases photosynthesis, lowers osmotic potential, and keeps plants in a state primed for freezing protection [41]. 

**Figure 5:**
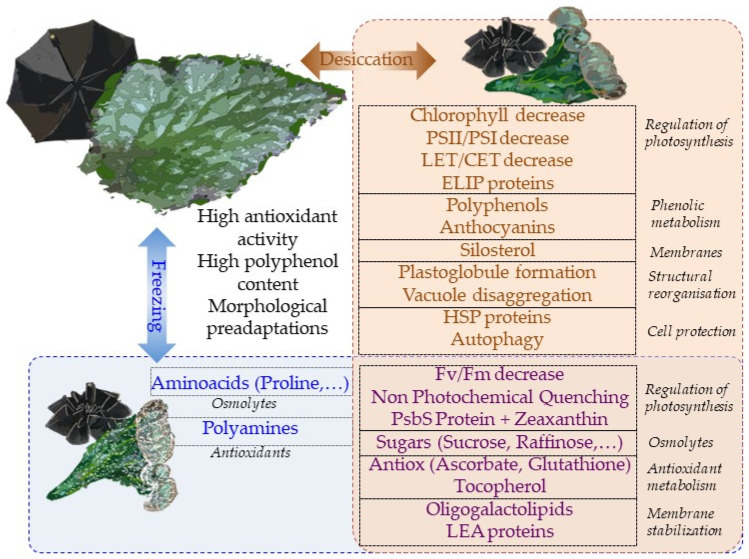
A summary of the main mechanisms of tolerance to desiccation and freezing in gesneriads. Brown box includes the responses to desiccation while blue box contains the mechanisms activated in response to freezing. The intersection between both boxes contains the mechanisms which are common to both stresses. Constitutive traits of gesneriads that might favor cross-tolerance are shown out of both boxes. The foldable umbrella analogy is depicted in the background.

Overall, most physiological mechanisms are shared in terms of the responses to desiccation and low-temperature stresses (Figure 5). This is perhaps the reason why tertiary paleotropical relict gesneriads were able to survive in sheltered habitats of southern Europe during the quaternary glaciations. The question of whether freezing tolerance is a constitutive trait in resurrection gesneriads or evolved in temperate species as a result of climate cooling deserves further studies.

## 4. Conclusions

Full understanding of the desiccation tolerance physiological response is still missing. The bulk of scientific studies on DT gesneriads have mainly researched leaf tissues, while the role of roots and root-associated soil microbiota has not been taken into consideration. Moreover, resurrection plants have usually been researched using physiological, transcriptomic, and metabolomic methods, mainly focusing on protection mechanisms during the desiccation phase, and the scarce studies on the rehydration phase are usually centered on the later stages of rehydration [41]. There is no record of whether morphological leaf preadaptations spread in both resurrection and non-resurrection members of the family are reflected in higher basal levels or greater numbers of coding domains of molecules that intervene in the different adaptation strategies. Similarly, the critical point at which rehydration starts has not been fully documented [106]. Indeed, even though water triggers the metabolic adaptation, changes in the dynamic state of water’s molecular structure and aquaphotomics are still emerging fields of study concerning resurrection species [134]. Equally, data on protein and DNA integrity maintenance and repair and mitochondrial functioning are also scarce despite their importance [105,135]. There is little information on the response of DT plants to other abiotic stresses, such as low temperatures, despite their similar physiological responses. 

The descriptions of the physiological, geographical, and morphological patterns provide evidence that habitat, growth form, morphology, and phylogenetic origin have jointly shaped gesneriads at the beginning of their evolution, making them more resilient to drought stress. In fact, apart from the DT strategy, other resistance growth forms, such as leaf abscission and geophytism, have also successfully developed. On the whole, the plasticity and adaptability of gesneriads are indisputable, and the whole Gesneriaceae family seems predisposed to endure dry circumstances in a great variety of habitats and climates. Thus, they could be the source of resurrection and double-tolerant species worldwide. In fact, the identified common traits have allowed us to propose tentative new double-tolerant/DT gesneriads that can make DT species more easily accessible and help broaden the knowledge of the mechanisms listed above.

## Figures and Tables

**Table 1:** Tentative new double-tolerant/DT species proposed according to the phylogenetic, geographical, and morphological patterns already described.

Subtribe	Documented DT Species	Tentative Double-Tolerant/DT Species
Streptocarpinae	*Streptocarpus revivescens*	*Streptocarpus rexii* *Streptocarpus meyeri* *Streptocarpus baudertii* *Streptocarpus montigena* *Streptocarpus rhodesianus*
Corallodiscinae	-	*Corallodiscus kingianus* *Corallodiscus cooperi* *Corallodiscus bhutanicus*
Didymocarpinae	*Oreocharis billburttii* *Oreocharis primuloides* *Oreocharis mileensis*	*Oreocharis pankaiyuae* *Oreocharis mairei* *Oreocharis ovatilobata* *Oreocharis flavovirens* *Oreocharis muscicola* *Oreocharis blepharophylla* *Oreocharis delavayi* *Oreocharis ninglangensis* *Oreocharis crispata* *Oreocharis magnidens* *Oreocharis stewardii*
Didymocarpinae	-	*Henckelia incana* *Henckelia gambleana* *Henckelia fischeri* *Henckelia bracteata* *Henckelia wayanadensis* *Henckelia innominata*

## Data Availability

Not applicable.

## References

[1] Weber A., Clark J.L., Möller M. (2013). A New Formal Classification of Gesneriaceae. Selbyana.

[2] Marks R.A., Farrant J.M., Nicholas McLetchie D., VanBuren R. (2021). Unexplored Dimensions of Variability in Vegetative Desiccation Tolerance. Am. J. Bot..

[3] Weber A., Kulbitzki K. (2004). Gesneriaceae. The Families and Genera of Vascular Plants.

[4] Verhoeven A., García-Plazaola J.I., Fernández-Marín B. (2018). Shared Mechanisms of Photoprotection in Photosynthetic Organisms Tolerant to Desiccation or to Low Temperature. Environ. Exp. Bot..

[5] Tebele S.M., Marks R.A., Farrant J.M. (2021). Two Decades of Desiccation Biology: A Systematic Review of the Best Studied Angiosperm Resurrection Plants. Plants.

[6] Clark J.L., Funke M.M., Duffy A.M., Smith J.F. (2012). Phylogeny of a Neotropical Clade in the Gesneriaceae: More Tales of Convergent Evolution. Int. J. Plant Sci..

[7] Serrano-Serrano M.L., Rolland J., Clark J.L., Salamin N., Perret M. (2017). Hummingbird Pollination and the Diversification of Angiosperms: An Old and Successful Association in Gesneriaceae. Proc. R. Soc. B Biol. Sci..

[8] Roalson E.H., Roberts W.R. (2016). Distinct Processes Drive Diversification in Different Clades of Gesneriaceae. Syst. Biol..

[9] Ogutcen E., Christe C., Nishii K., Salamin N., Möller M., Perret M. (2021). Phylogenomics of Gesneriaceae Using Targeted Capture of Nuclear Genes. Mol. Phylogenet. Evol..

[10] Möller M., Pfosser M., Jang C.G., Mayer V., Clark A., Hollingsworth M.L., Barfuss M.H.J., Wang Y.Z., Kiehn M., Weber A. (2009). A Preliminary Phylogeny of the “didymocarpoid Gesneriaceae” Based on Three Molecular Data Sets: Incongruence with Available Tribal Classifications. Am. J. Bot..

[11] Burtt B.L., Wiehler H. (1995). Classification of the Family Gesneriaceae. Gesneriana.

[12] Xu W., Guo J., Pan B., Zhang Q., Liu Y. (2017). Diversity and Distribution of Gesneriaceae in China. Guihaia.

[13] Möller M., Wei Y.G., Wen F., Clark J.L., Weber A. (2016). You Win Some You Lose Some: Updated Generic Delimitations and Classification of Gesneriaceae-Implications for the Family in China. Guihaia.

[14] Weber A., Middleton D.J., Clark J.L., Möller M. (2020). Keys to the Infrafamilial Taxa and Genera of Gesneriaceae. J. Indian Assoc. Angiosperm Taxon..

[15] Tan K., Lu T., Ren M.X. (2020). Biogeography and Evolution of Asian Gesneriaceae Based on Updated Taxonomy. PhytoKeys.

[16] Middleton D.J., Atkins H., Truong L.H., Nishii K., Möller M. (2014). Billolivia, a New Genus of Gesneriaceae from Vietnam with Five New Species. Phytotaxa.

[17] Wen F., Xin Z.B., Fu L.F., Li S., Su L.Y., Maciejewski S., Huang Z.J., Van Do T., Wei Y.G. (2020). Michaelmoelleria (Gesneriaceae), a New Lithophilous Dwelling Genus and Species with Zigzag Corolla Tube from Southern Vietnam. PhytoKeys.

[18] Middleton D.J., Nishii K., Puglisi C., Forrest L.L., Möller M. (2015). Chayamaritia (Gesneriaceae: Didymocarpoideae), a New Genus from Southeast Asia on JSTOR. Plant Syst. Evol..

[19] Möller M., Chen W.-H., Shui Y.-M., Atkins H., Middleton D.J. (2014). A New Genus of Gesneriaceae in China and the Transfer of Briggsia Species to Other Genera. Gard. Bull. Singap..

[20] Puglisi C., Middleton D.J. (2017). A Revision of *Microchirita* (Gesneriaceae) in Thailand. Gard. Bull. Singap..

[21] Puglisi C., Yao T.L., Milne R., Möller M., Middleton D.J. (2016). Generic Recircumscription in the Loxocarpinae (Gesneriaceae), as Inferred by Phylogenetic and Morphological Data. Taxon.

[22] Middleton D.J., Khew G.S., Poopath M., Möller M., Puglisi C. (2018). *Rachunia cymbiformis*, a New Genus and Species of Gesneriaceae from Thailand. Nord. J. Bot..

[23] Middleton D.J., Triboun P. (2013). A New Species of *Somrania* (Gesneriaceae) from Thailand. Bull. Singap..

[24] Puglisi C., Middleton D.J. (2017). A Revision of *Damrongia* (Gesneriaceae) in Thailand. Thai For. Bull..

[25] Möller M., Nishii K., Atkins H.J., Kong H.H., Kang M., Wei Y.G., Wen F., Hong X., Middleton D.J. (2016). An Expansion of the Genus *Deinostigma* (Gesneriaceae). Gard. Bull. Singap..

[26] Wen F., Li S., Xin Z., Fu L.F., Hong X., Cai L., Qin J.Q., Pan B., Pan F.Z., Wei Y.G. (2019). The Latest List of Gesneriaceae in China under the New Chinese Nomenclature. Guangxi Sci..

[27] Nishii K., Hughes M., Briggs M., Haston E., Christie F., DeVilliers M.J., Hanekom T., Roos W.G., Bellstedt D.U., Möller M. (2015). *Streptocarpus* Redefined to Include All Afro-Malagasy Gesneriaceae: Molecular Phylogenies Prove Congruent with Geographical Distribution and Basic Chromosome Numbers and Uncover Remarkable Morphological Homoplasies. Taxon.

[28] Middleton D.J., Weber A., Yao T.L., Sontag S., Möller M. (2013). The Current Status of the Species Hitherto Assigned to *Henckelia* (Gesneriaceae). Edinb. J. Bot..

[29] Xin D., Zhiang H., Hongxin W., Xiaogang W., Tingyun K. (2000). Effects of Dehydration and Rehydration on Photosynthesis of Detached Leaves of the Resurrective Plant *Boea hygrometrica*. Acta Bot. Sin..

[30] Wang Y., Liu K., Bi D., Zhou S., Shao J. (2018). Molecular Phylogeography of East Asian *Boea clarkeana* (Gesneriaceae) in Relation to Habitat Restriction. PLoS ONE.

[31] Maria Sgherri C.L., Loggini B., Bochicchio A., Navari-Izzo F. (1994). Antioxidant System in *Boea hygroscopica*: Changes in Response to Desiccation and Rehydration. Phytochemistry.

[32] Gray B., Tropical Herbarium A., Cook University J., Qld S. (2021). *Boea resupinata* Zich & B.Gray (Gesneriaceae), a New Species from Cape York Peninsula, Queensland, Australia. Austrobaileya.

[33] Zhao H., Liu H., Yu H., Hu Y., Gao Y., Li Z., Lin Z. (2000). Cloning and Expression Pattern of a Dehydrin-Like*BDN1* Gene from Drought-Tolerant *Boea crassifolia* Hemsl. Chin. Sci. Bull..

[34] Huang W., Yang S.J., Zhang S.B., Zhang J.L., Cao K.F. (2012). Cyclic Electron Flow Plays an Important Role in Photoprotection for the Resurrection Plant *Paraboea rufescens* under Drought Stress. Planta.

[35] Djilianov D.L., Moyankova D.P., Mladenov P.V. (2016). The Mediterranean: A Cradle of the Resurrection Plants in Europe. Phytol. Balc..

[36] Rakić T., Lazarević M., Jovanović Ž.S., Radović S., Siljak-Yakovlev S., Stevanović B., Stevanović V. (2014). Resurrection Plants of the Genus *Ramonda*: Prospective Survival Strategies-Unlock Further Capacity of Adaptation, or Embark on the Path of Evolution?. Front. Plant Sci..

[37] Muller J., Sprenger N., Bortlik K., Boiler T., Wiemken Muller A., Bortlik N., Wiemken T., Muller J., Sprenger N., Bortlik K. (1997). Desiccation Increases Sucrose Levels in *Ramonda* and *Haberlea*, Two Genera of Resurrection Plants in the Gesneriaceae. Physiol. Plant.

[38] Gaff D.F., Oliver M. (2013). The Evolution of Desiccation Tolerance in Angiosperm Plants: A rare yet common phenomenon. Funct. Plant Biol..

[39] Li A., Wang D., Yu B., Yu X., Li W. (2014). Maintenance or Collapse: Responses of Extraplastidic Membrane Lipid Composition to Desiccation in the Resurrection Plant *Paraisometrum mileense*. PLoS ONE.

[40] Fernández-Marín B., Neuner G., Kuprian E., Laza J.M., García-Plazaola J.I., Verhoeven A. (2018). First Evidence of Freezing Tolerance in a Resurrection Plant: Insights into Molecular Mobility and Zeaxanthin Synthesis in the Dark. Physiol. Plant.

[41] Benina M., Obata T., Mehterov N., Ivanov I., Petrov V., Toneva V., Fernie A.R., Gechev T.S. (2013). Comparative Metabolic Profiling of *Haberlea rhodopensis*, *Thellungiella halophyla*, and *Arabidopsis thaliana* Exposed to Low Temperature. Front. Plant Sci..

[42] Morley R.J. (2003). Interplate Dispersal Paths for Megathermal Angiosperms. Perspect. Plant Ecol. Evol. Syst..

[43] Perret M., Chautems A., de Araujo A.O., Salamin N. (2013). Temporal and Spatial Origin of Gesneriaceae in the New World Inferred from Plastid DNA Sequences. Bot. J. Linn. Soc..

[44] Woo V.L., Funke M.M., Smith J.F., Lockhart P.J., Garnock-Jones P.J. (2011). New World Origins of Southwest Pacific Gesneriaceae: Multiple Movements Across and Within the South Pacific. Int. J. Plant Sci..

[45] Petrova G., Moyankova D., Nishii K., Forrest L., Tsiripidis I., Drouzas A.D., Djilianov D., Möller M. (2015). The European Paleoendemic *Haberlea rhodopensis* (Gesneriaceae) Has an Oligocene Origin and a Pleistocene Diversification and Occurs in a Long-Persisting Refugial Area in Southeastern Europe. Int. J. Plant Sci..

[46] Hultén E., Fries M. (1986). Atlas of North European Vascular Plants: North of the Tropic of Cancer.

[47] Bettin O., Cornejo C., Edwards P.J., Holderegger R. (2007). Phylogeography of the High Alpine Plant *Senecio halleri* (Asteraceae) in the European Alps: In Situ Glacial Survival with Postglacial Stepwise Dispersal into Peripheral Areas. Mol. Ecol..

[48] Milne R., Abbott R.J. (2002). The Origin and Evolution of Tertiary Relict Floras. Adv. Bot. Res..

[49] Skog L.E. (1976). A Study of the Tribe Gesneriaceae, with a Revision of *Gesneria* (Gesneriaceae-Gesnerioideae). Smithson. Contrib. Bot..

[50] Martén-Rodríguez S., Fenster C.B., Agnarsson I., Skog L.E., Zimmer E.A. (2010). Evolutionary Breakdown of Pollination Specialization in a Caribbean Plant Radiation. New Phytol..

[51] Perret M., Chautems A., Spichiger R. (2006). Dispersal-Vicariance Analyses in the Tribe Sinningiaeae (Gesneriaceae): A Clue to Understanding Biogeographical History of the Brazilian Atlanric Forest. Ann. Mo. Bot. Gard..

[52] Clements R., Sodhi N.S., Schilthuizen M., Hg P.K.L. (2006). Limestone Karsts of Southeast Asia: Imperiled Arks of Biodiversity. Bioscience.

[53] Porembski S., Barthlott W. (2000). Granitic and Gneissic Outcrops (Inselbergs) as Centers of Diversity for Desiccation-Tolerant Vascular Plants. Plant Ecol..

[54] Alejo-Jacuinde G., Herrera-Estrella L. (2022). Exploring the High Variability of Vegetative Desiccation Tolerance in Pteridophytes. Plants.

[55] Liu C., Huang Y., Wu F., Liu W., Ning Y., Huang Z., Tang S., Liang Y. (2021). Plant Adaptability in Karst Regions. J. Plant Res..

[56] Burke A. (2002). Island–Matrix Relationships in Nama Karoo Inselberg Landscapes Part II: Are Some Inselbergs Better Sources than Others?. Plant Ecol..

[57] Smith S.A., Beaulieu J.M. (2009). Life History Influences Rates of Climatic Niche Evolution in Flowering Plants. Proc. R. Soc. B Biol. Sci..

[58] Möller M., Cronk Q.C.B. (2001). Evolution of Morphological Novelty: A Phylogenetic Analysis of Growth Patterns in *Streptocarpus* (Gesneriaceae). Evolution.

[59] Rafsanjani A., Brulé V., Western T.L., Pasini D. (2015). Hydro-Responsive Curling of the Resurrection Plant *Selaginella lepidophylla*. Sci. Rep..

[60] Körner C. (2003). Alpine Plant Life: Functional Plant Ecology of High Mountain Ecosystems.

[61] Sklenář P., Kučerová A., Macková J., Romoleroux K. (2018). Temperature Microclimates of Plants in a Tropical Alpine Environment: How Much Does Growth Form Matter?. Arct. Antarct. Alp. Res..

[62] Werk K.S., Ehleringer J., Forseth I.N., Cook C.S. (1983). Photosynthetic Characteristics of Sonoran Desert Winter Annuals. Oecologia.

[63] Sakai A., Larcher W. (1987). Frost Survival of Plants: Responses and Adaptation to Freezing Stress.

[64] Alpert P. (2006). Constraints of Tolerance: Why Are Desiccation-Tolerant Organisms so Small or Rare?. J. Exp. Biol..

[65] Asami P., Mundree S., Williams B. (2018). Saving for a Rainy Day: Control of Energy Needs in Resurrection Plants. Plant Sci..

[66] Squeo F.A., Rada F., Azocar A., Goldstein G. (1991). Freezing Tolerance and Avoidance in High Tropical Andean Plants: Is It Equally Represented in Species with Different Plant Height?. Oecologia.

[67] Cruz-Maldonado N., Weemstra M., Jiménez L., Roumet C., Angeles G., Barois I., de los Santos M., Morales-Martinez M.A., Palestina R.A., Rey H. (2021). Aboveground-Trait Variations in 11 (Sub)Alpine Plants along a 1000-m Elevation Gradient in Tropical Mexico. Alp. Bot..

[68] Leon-Garcia I.V., Lasso E. (2019). High Heat Tolerance in Plants from the Andean Highlands: Implications for Paramos in a Warmer World. PLoS ONE.

[69] Scheepens J.F., Frei E.S., Stöcklin J. (2010). Genotypic and Environmental Variation in Specific Leaf Area in a Widespread Alpine Plant after Transplantation to Different Altitudes. Oecologia.

[70] Beck E., Schulze E.D., Senser M., Scheibe R. (1984). Equilibrium Freezing of Leaf Water and Extracellular Ice Formation in Afroalpine ‘Giant Rosette’ Plants. Planta.

[71] Melcher P.J., Goldstein G., Meinzer F.C., Minyard B., Giambelluca T.W., Loope L.L. (1994). Determinants of Thermal Balance in the Hawaiian Giant Rosette Plant, *Argyroxiphium sandwicense*. Oecologia.

[72] McKown A.D., Cochard H., Sack L. (2010). Decoding Leaf Hydraulics with a Spatially Explicit Model: Principles of Venation Architecture and Implications for Its Evolution. Am. Nat..

[73] Niklas K.J. (1999). A Mechanical Perspective on Foliage Leaf Form and Function. New Phytol..

[74] Kehr J., Buhtz A. (2008). Long Distance Transport and Movement of RNA through the Phloem. J. Exp. Bot..

[75] Sack L., Frole K. (2006). Leaf Structural Diversity Is Related to Hydraulic Capacity in Tropical Rain Forest Trees. Ecology.

[76] Givnish T. (1987). Comparative Studies of Leaf from: Assessing the Relative Roles of Selective Preassures and Phylogenetic Constraints. New Phytol..

[77] Fonseca C.R., Overton J.M.C., Collins B., Westoby M. (2000). Shifts in Trait-Combinations along Rainfall and Phosphorus Gradients. J. Ecol..

[78] Davis S.D., Sperry J.S., Hacke U.G. (1999). The Relationship between Xylem Conduit Diameter and Cavitation Caused by Freezing. Am. J. Bot..

[79] Baas P., Ewers F.W., Davis S.D., Wheeler E.A., Hemsley A.R., Poole I. (2004). Evolution of Xylem Physiology. The Evolution of Plant Physiology.

[80] Scoffoni C., Rawls M., Mckown A., Cochard H., Sack L. (2011). Decline of Leaf Hydraulic Conductance with Dehydration: Relationship to Leaf Size and Venation Architecture. Plant Physiol..

[81] Sack L., Dietrich E.M., Streeter C.M., Sánchez-Gómez D., Holbrook N.M. (2008). Leaf Palmate Venation and Vascular Redundancy Confer Tolerance of Hydraulic Disruption. Proc. Natl. Acad. Sci. USA.

[82] Ichie T., Inoue Y., Takahashi N., Kamiya K., Kenzo T. (2016). Ecological Distribution of Leaf Stomata and Trichomes among Tree Species in a Malaysian Lowland Tropical Rain Forest. J. Plant Res..

[83] Dahlin R.M., Brick M.A., Barry J., Dahlin O.R.M., Brick M.A., Ogg J.B. (1992). Characterization and Density of Trichomes on Three Common Bean Cultivars. Econ. Bot..

[84] Benz B.W., Martin C.E. (2006). Foliar Trichomes, Boundary Layers, and Gas Exchange in 12 Species of Epiphytic *Tillandsia* (Bromeliaceae). J. Plant Physiol..

[85] Agati G., Tattini M. (2010). Multiple Functional Roles of Flavonoids in Photoprotection on JSTOR. New Phytol..

[86] Gutiérrez-Alcalá G., Gotor C., Meyer A.J., Fricker M., Vega J.M., Romero L.C. (2000). Glutathione Biosynthesis in *Arabidopsis* Trichome Cells. Proc. Natl. Acad. Sci. USA.

[87] Hauser M.T. (2014). Molecular Basis of Natural Variation and Environmental Control of Trichome Patterning. Front. Plant Sci..

[88] Prozherina N., Freiwald V., Rousi M., Oksanen E. (2003). Interactive Effect of Springtime Frost and Elevated Ozone on Early Growth, Foliar Injuries and Leaf Structure of Birch (*Betula pendula*). New Phytol..

[89] Tian D., Peiffer M., de Moraes C.M., Felton G.W. (2014). Roles of Ethylene and Jasmonic Acid in Systemic Induced Defense in Tomato (*Solanum lycopersicum*) against *Helicoverpa zea*. Planta.

[90] Ning P., Wang J., Zhou Y., Gao L., Wang J., Gong C. (2016). Adaptional Evolution of Trichome in *Caragana korshinskii* to Natural Drought Stress on the Loess Plateau, China. Ecol. Evol..

[91] Yan A., Pan J., An L., Gan Y., Feng H. (2012). The Responses of Trichome Mutants to Enhanced Ultraviolet-B Radiation in *Arabidopsis thaliana*. J. Photochem. Photobiol. B.

[92] Schuepp P.H. (1993). Leaf Boundary Layers. New Phytol..

[93] Lacey M. (2019). From Fossils to Physiology: Testing the Functional Significance of Leaf Shape.

[94] Willson C.J., Manos P.S., Jackson R.B. (2008). Hydraulic Traits Are Influenced by Phylogenetic History in the Drought-Resistant, Invasive Genus *Juniperus* (Cupressaceae). Am. J. Bot..

[95] Moore J.P., Lindsey G.G., Farrant J.M., Brandt W.F. (2007). An Overview of the Biology of the Desiccation-Tolerant Resurrection Plant *Myrothamnus flabellifolia*. Ann. Bot..

[96] Kampowski T., Demandt S., Poppinga S., Speck T. (2018). Kinematical, Structural and Mechanical Adaptations to Desiccation in Poikilohydric *Ramonda myconi* (Gesneriaceae). Front. Plant Sci..

[97] Vieira E.A., Silva K.R., Oriani A., Moro C.F., Braga M.R. (2017). Mechanisms of Desiccation Tolerance in the Bromeliad *Pitcairnia burchellii* Mez: Biochemical Adjustments and Structural Changes. Plant Physiol. Biochem..

[98] Heilmeier H., Hartung W., Lüttge U., Beck E., Bartel D. (2011). Chamaegigas Intrepidus DINTER: An Aquatic Poikilohydric Angiosperm That Is Perfectly Adapted to Its Complex and Extreme Environmental Conditions. Plant Desiccation Tolerance. Ecological Studies.

[99] Gaff D.F. (1986). Desiccation Tolerant ‘Resurrection’ Grasses from Kenya and West Africa. Oecologia.

[100] Shen Y., Tang M.J., Hu Y.L., Lin Z.P. (2004). Isolation and Characterization of a Dehydrin-like Gene from Drought-Tolerant *Boea crassifolia*. Plant Sci..

[101] Fernández-Marín B., Nadal M., Gago J., Fernie A.R., López-Pozo M., Artetxe U., García-Plazaola J.I., Verhoeven A. (2020). Born to Revive: Molecular and Physiological Mechanisms of Double Tolerance in a Paleotropical and Resurrection Plant. New Phytol..

[102] Jovanović Ž., Rakić T., Stevanović B., Radović S. (2011). Characterization of Oxidative and Antioxidative Events during Dehydration and Rehydration of Resurrection Plant *Ramonda nathaliae*. Plant Growth Regul..

[103] Liu J., Moyankova D., Djilianov D., Deng X. (2019). Common and Specific Mechanisms of Desiccation Tolerance in Two Gesneriaceae Resurrection Plants. Multiomics Evidences. Front. Plant Sci..

[104] Drazic G., Mihailovic N., Stevanovic B. (1999). Chlorophyll Metabolism in Leaves of Higher Poikilohydric Plants *Ramonda serbica* Panč, and *Ramonda nathaliae* Panč, et Petrov. during Dehydration and Rehydration. J. Plant Physiol..

[105] Liu J., Moyankova D., Lin C.T., Mladenov P., Sun R.Z., Djilianov D., Deng X. (2018). Transcriptome Reprogramming during Severe Dehydration Contributes to Physiological and Metabolic Changes in the Resurrection Plant *Haberlea rhodopensis*. BMC Plant Biol..

[106] Georgieva K., Mihailova G., Velitchkova M., Popova A. (2020). Recovery of Photosynthetic Activity of Resurrection Plant *Haberlea rhodopensis* from Drought-and Freezing-Induced Desiccation. Photosynthetica.

[107] Mladenov P., Finazzi G., Bligny R., Moyankova D., Zasheva D., Boisson A.M., Brugière S., Krasteva V., Alipieva K., Simova S. (2015). In Vivo Spectroscopy and NMR Metabolite Fingerprinting Approaches to Connect the Dynamics of Photosynthetic and Metabolic Phenotypes in Resurrection Plant *Haberlea rhodopensis* during Desiccation and Recovery. Front. Plant Sci..

[108] Asada K. (2006). Production and Scavenging of Reactive Oxygen Species in Chloroplasts and Their Functions. Plant Physiol..

[109] Oliver M.J., Farrant J.M., Hilhorst H.W.M., Mundree S., Williams B., Bewley J.D. (2020). Desiccation Tolerance: Avoiding Cellular Damage during Drying and Rehydration. Annu. Rev. Plant Biol..

[110] Georgieva K., Rapparini F., Bertazza G., Mihailova G., Sárvári É., Solti Á., Keresztes Á. (2017). Alterations in the Sugar Metabolism and in the Vacuolar System of Mesophyll Cells Contribute to the Desiccation Tolerance of *Haberlea rhodopensis* Ecotypes. Protoplasma.

[111] Cañigueral S., Salvía M.J., Vila R., Iglesias J., Virgili A., Parella T. (1996). New Polyphenol Glycosides from *Ramonda myconi*. J. Nat. Prod..

[112] Jensen S.R. (1996). Caffeoyl Phenylethanoid Glycosides in *Sanango racemosum* and in the Gesneriaceae. Phytochemistry.

[113] Feng W.S., Li Y.J., Zheng X.K., Wang Y.Z., Su F.Y., Pei Y.Y. (2011). Two New C-Glycosylflavones from *Boea hygrometrica*. J. Asian Nat. Prod. Res..

[114] Georgieva K., Dagnon S., Gesheva E., Bojilov D., Mihailova G., Doncheva S. (2017). Antioxidant Defense during Desiccation of the Resurrection Plant *Haberlea rhodopensis*. Plant Physiol. Biochem..

[115] Gechev T.S., Benina M., Obata T., Tohge T., Sujeeth N., Minkov I., Hille J., Temanni M.R., Marriott A.S., Bergström E. (2013). Molecular Mechanisms of Desiccation Tolerance in the Resurrection Glacial Relic *Haberlea rhodopensis*. Cell. Mol. Life Sci..

[116] Mladenov P.V., Zasheva D., Planchon S., Leclercq C., Falconet D., Moyet L., Brugière S., Moyankova D., Tchorbadjieva M., Ferro M. (2022). Proteomics Evidence of a Systemic Response to Desiccation in the Resurrection Plant *Haberlea rhodopensis*. SSRN Electron. J..

[117] Quartacci M.F., Glišić O., Stevanović B., Navari-Izzo F. (2002). Plasma Membrane Lipids in the Resurrection Plant *Ramonda serbica* Following Dehydration and Rehydration. J. Exp. Bot..

[118] Moyankova D., Mladenov P., Berkov S., Peshev D., Georgieva D., Djilianov D. (2014). Metabolic Profiling of the Resurrection Plant *Haberlea rhodopensis* during Desiccation and Recovery. Physiol. Plant.

[119] Navari-Izzo F., Ricci F., Vazzana C., Quartacci M.F. (1995). Unusual Composition of Thylakoid Membranes of the Resurrection Plant *Boea hygroscopica*: Changes in Lipids upon Dehydration and Rehydration. Physiol. Plant.

[120] Zhu Y., Wang B., Phillips J., Zhang Z.N., Du H., Xu T., Huang L.C., Zhang X.F., Xu G.H., Li W.L. (2015). Global Transcriptome Analysis Reveals Acclimation-Primed Processes Involved in the Acquisition of Desiccation Tolerance in *Boea hygrometrica*. Plant Cell Physiol..

[121] Pinnola A., Bassi R. (2018). Molecular Mechanisms Involved in Plant Photoprotection. Biochem. Soc. Trans..

[122] Mihailova G., Vasileva I., Gigova L., Gesheva E., Simova-Stoilova L., Georgieva K. (2022). Antioxidant Defense during Recovery of Resurrection *Haberlea rhodopensis* from Drought- and Freezing-Induced Desiccation. Plants.

[123] Mitra J., Xu G., Wang B., Li M., Deng X. (2013). Understanding Desiccation Tolerance Using the Resurrection Plant *Boea hygrometrica* as a Model System. Front. Plant Sci..

[124] Mihailova G., Kocheva K., Goltsev V., Kalaji H.M., Georgieva K. (2018). Application of a Diffusion Model to Measure Ion Leakage of Resurrection Plant Leaves Undergoing Desiccation. Plant Physiol. Biochem..

[125] Farrant J.M., Cooper K., Nell H. (2000). Desiccation Tolerance. Plant Stress Physiology.

[126] Georgieva K., Sárvári É., Keresztes Á. (2010). Protection of Thylakoids against Combined Light and Drought by a Lumenal Substance in the Resurrection Plant *Haberlea rhodopensis*. Ann. Bot..

[127] Djilianov D., Ivanov S., Moyankova D., Miteva L., Kirova E., Alexieva V., Joudi M., Peshev D., van den Ende W. (2011). Sugar Ratios, Glutathione Redox Status and Phenols in the Resurrection Species *Haberlea rhodopensis* and the Closely Related Non-Resurrection Species *Chirita eberhardtii*. Plant Biol..

[128] Petrov V., Hille J., Mueller-Roeber B., Gechev T.S. (2015). ROS-Mediated Abiotic Stress-Induced Programmed Cell Death in Plants. Front. Plant Sci..

[129] Kappen V.L. (1966). Sucht an Blättern Einiger Farne Und von *Ramonda myconi*. Flora.

[130] Georgieva K., Mihailova G., Gigova L., Dagnon S., Simova-Stoilova L., Velitchkova M. (2021). The Role of Antioxidant Defense in Freezing Tolerance of Resurrection Plant *Haberlea rhodopensis*. Physiol. Mol. Biol. Plants.

[131] Mihailova G., Solti Á., Sárvári É., Keresztes Á., Rapparini F., Velitchkova M., Simova-Stoilova L., Aleksandrov V., Georgieva K. (2020). Freezing Tolerance of Photosynthetic Apparatus in the Homoiochlorophyllous Resurrection Plant *Haberlea rhodopensis*. Environ. Exp. Bot..

[132] Mihailova G., Christov N.K., Sárvári É., Solti Á., Hembrom R., Solymosi K., Keresztes Á., Velitchkova M., Popova A.V., Simova-Stoilova L. (2022). Reactivation of the Photosynthetic Apparatus of Resurrection Plant *Haberlea rhodopensis* during the Early Phase of Recovery from Drought- and Freezing-Induced Desiccation. Plants.

[133] Georgieva K., Popova A.V., Mihailova G., Ivanov A.G., Velitchkova M. (2022). Limiting Steps and the Contribution of Alternative Electron Flow Pathways in the Recovery of the Photosynthetic Functions after Freezing-Induced Desiccation of *Haberlea rhodopensis*. Photosynthetica.

[134] Kuroki S., Tsenkova R., Moyankova D., Muncan J., Morita H., Atanassova S., Djilianov D. (2019). Water Molecular Structure Underpins Extreme Desiccation Tolerance of the Resurrection Plant *Haberlea rhodopensis*. Sci. Rep..

[135] Ivanova A., O′Leary B., Signorelli S., Falconet D., Moyankova D., Whelan J., Djilianov D., Murcha M.W. (2022). Mitochondrial Activity and Biogenesis during Resurrection of *Haberlea rhodopensis*. New Phytol..

